# Genetic differentiation in red‐bellied piranha populations (*Pygocentrus nattereri*, Kner, 1858) from the Solimões‐Amazonas River

**DOI:** 10.1002/ece3.2195

**Published:** 2016-05-24

**Authors:** Carlos Henrique dos A. dos Santos, Carolina S. de Sá Leitão, Maria de N. Paula‐Silva, Vera Maria F. Almeida‐Val

**Affiliations:** ^1^ Laboratório de Ecofisiologia e Evolução MolecularInstituto Nacional de Pesquisas da AmazôniaAv. André Araújo 2936, Aleixo69060‐001ManausBrasil; ^2^ Laboratório de Genética Aplicada à Aquicultura & Biologia MolecularUniversidade Nilton LinsPrograma de Pós‐Graduação em AquiculturaAv. Professor Nilton Lins 3259, Parque das Laranjeiras69058‐030ManausBrasil

**Keywords:** Biological populations, gene flow, genetic diversity, geographical distance, management

## Abstract

Red‐bellied piranhas (*Pygocentrus nattereri*) are widely caught with different intensities throughout the region of Solimões‐Amazonas River by local fishermen. Thus, the management of this resource is performed in the absence of any information on its genetic stock. *P. nattereri* is a voracious predator and widely distributed in the Neotropical region, and it is found in other regions of American continent. However, information about genetic variability and structure of wild populations of red‐bellied piranha is unavailable. Here, we describe the levels of genetic diversity and genetic structure of red‐bellied piranha populations collected at different locations of Solimões‐Amazonas River system. We collected 234 red‐bellied piranhas and analyzed throughout eight microsatellite markers. We identified high genetic diversity within populations, although the populations of lakes ANA, ARA, and MAR have shown some decrease in their genetic variability, indicating overfishing at these communities. Was identified the existence of two biological populations when the analysis was taken altogether at the lakes of Solimões‐Amazonas River system, with significant genetic differentiation between them. The red‐bellied piranha populations presented limited gene flow between two groups of populations, which were explained by geographical distance between these lakes. However, high level of gene flow was observed between the lakes within of the biological populations. We have identified high divergence between the Catalão subpopulation and all other subpopulations. We suggest the creation of sustainable reserve for lakes near the city of Manaus to better manage and protect this species, whose populations suffer from both extractive and sport fishing.

## Introduction

In general, fish species are not genetically homogeneous, but structured in groups of individuals who are relatively isolated. The identification of genetically homogenous groups is the basic unit for fish management, conservation, and sustainable use of genetic resources (Laikre et al. 2005). Thus, the knowledge of these groups’ structure is vital to reduce the risk of wild stocks depletion (Ryman and Utter 1987). The level of structure in natural populations are, then, of great importance for the evolutionary potential of the target population (Waples 1998; Begga and Waldmanb 1999), and then, for increasing populations immediate viability if inbreeding depression, decline of biological population, or local extinction are taking place (Allendorf and Ryman 2002; Laikre et al. 2005). In such context, it is clear that the levels of genetic structure are the kind of information that supports strategies for management of exploited stocks (Ricker 1981). Thus, determining and identifying fish stocks are the important tasks to distinguish one population from a set of geographically distributed subpopulations whose demographic and genetic histories are largely independent of other groups and present some degree of reproductive isolation (Saila and Jones 1983; Waples 1998; Cadrin and Secor 2009).

The red‐bellied piranha, *Pygocentrus nattereri*, is one of the most abundant fish species living in the floodplain lakes (Várzea lakes) of the Solimões‐Amazonas river system (Merona and Bittencourt 1993; Saint‐Paul et al. 2000), occurring also in “igapós” (flooded forests) and margins of Rio Negro. Their biological and demographic characteristics suggest that this species is very well adapted to survive in the floodplain environments (Bittencourt 1994). *P. nattereri* is popularly known as a voracious and aggressive predator. However, it is widely consumed and marketed by local fisherman (Silva and Araújo‐Lima 2003). We must emphasize that the red‐bellied piranha is fairly wide distributed in South American basins, being found in the river basins of Orinoco (Machado‐Allison and Fink 1995), São Francisco, Paraná and Prata (Lowe‐McConnell 1987; Hubert and Renno 2006). In general, all piranhas’ species occur in freshwater systems of Neotropical region and belong to the Serrasalmidae family, whose main genera are *Serrasalmus* and *Pygocentrus* (Mirande 2010).

Freshwater ichthyofauna of the Amazon basin has a large number of species well adapted to survive highly dynamics (annual flood pulses), warm and hypoxic waters (see the term Flood Pulses by Junk et al. 1989; Ruffino and Isaac 1995; Guyot et al. 1999), but are rather subject to the global changes (Costa and Foley 1999; Davidson et al. 2012). In recent years, the water bodies of the Amazon have suffered constant anthropic problems such as oil spills by petroleum vessels, construction of oil and gas pipelines, rivers obstructed by hydroelectric power plants, urban sewages launched in water, and overexploitation of fishery resources. Disturbances in this environment have changed many ecological niches of highly susceptible species, and this has directly influenced the entrance or recruitment of new individuals in adult stocks, producing significant changes in the size of wild populations.

Some authors have suggested that these changes have significantly influenced the contemporary gene flow of local population (Castric et al. 2001; Costello et al. 2003). Moreover, while the environmental heterogeneity has contributed to increase of genetic structure of wild populations, floodplains have acted as a homogeneous disperser of genetic diversity between the lakes of the Amazon basin (Hubert et al. 2007). Considering such complexity and variety of environments in the Amazon region, it is crucial to evaluate fish stocks at the genetic levels aiming to identify possible changes in the genetic patterns between subpopulations. This is the best way to improve fisheries management in the region and contribute to public policies aiming to increase preservation and conservation of overexploited stocks.

Genetic studies conducted with the piranhas have been focused merely in phylogenetic information within the family Serrasalmidae (Hubert et al. 2007; Mirande 2010), leaving some gaps in our knowledge about genetic diversity within and among populations in their natural environment. Thus, the main goal of this study is to provide valuable information of populations’ genetics of the red‐bellied piranha occurring in Amazon basin, intending to describe the occurrence of genetically differences caused by geographical distribution and environmental characteristics.

We hypothesize that panmixia occurs in the red‐bellied piranha population distributed along the floodplain lakes of the Solimões‐Amazonas river systems. To test this hypothesis, we used microsatellite markers to describe the levels of genetic differentiation and gene flow between its subpopulations, and their genetic diversity found within and between each subpopulation.

## Material and Methods

### Sample collection

**Figure 1:**
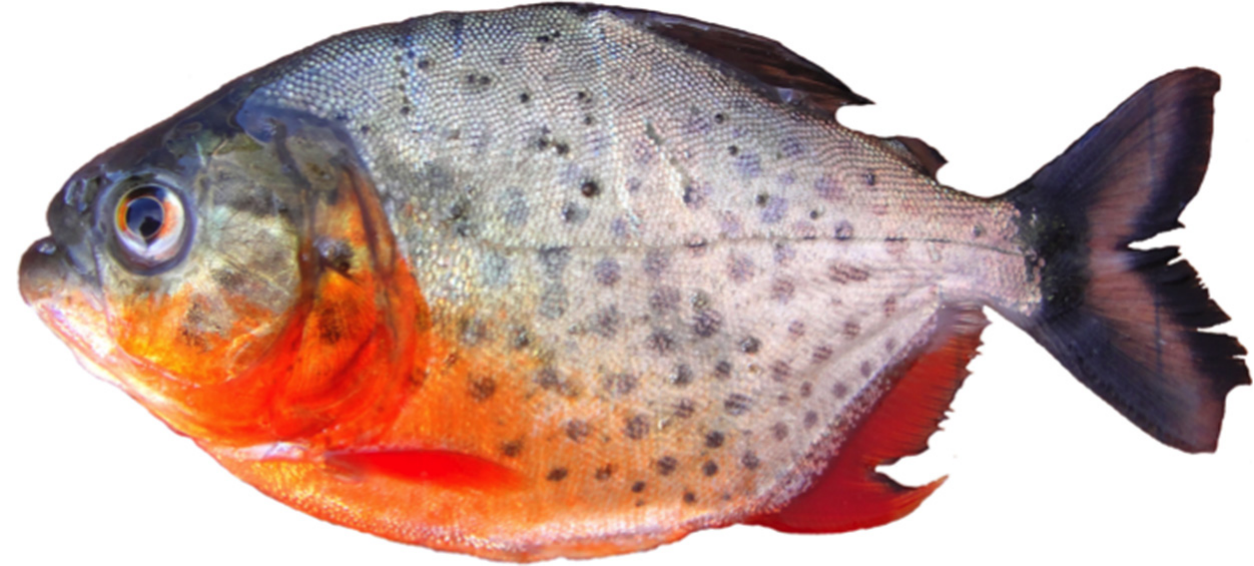
Specimen of *Pygocentrus nattereri*, a Neotropical fish from Solimões‐Amazonas River System in Brazil. Photograph credit: Waldir Heinrichs.

**Figure 2:**
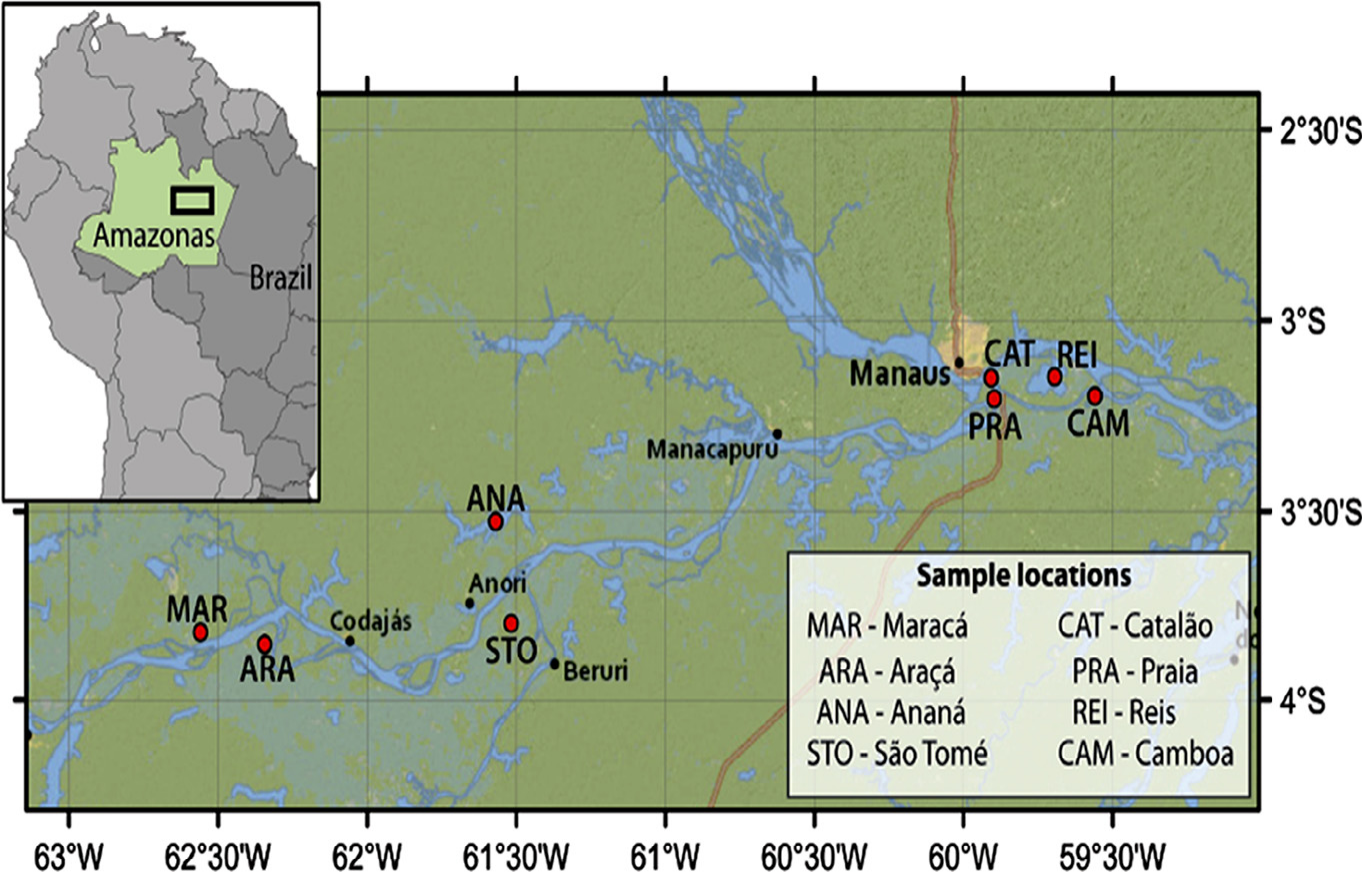
Locations of *Pygocentrus nattereri* sampling in the Solimões‐Amazonas River System. Color legend indicates the Lakes location in the map where populations were collected.

Specimens from wild populations of red‐bellied piranhas (Fig. 1) were caught in eight lakes of the Solimões‐Amazonas River system (Table 1 and Fig. 2). In total, we collected 234 red‐bellied piranhas and stored in ice. They were euthanized with a sharp blow to their head according to the Brazilian Guidelines for Ethical Care and Use of Animals (CONCEA 2013). The samples of white muscle tissue were stored in liquid nitrogen, until the total genomic DNA extraction was performed. Fish collection authorization was approved by SISBIO under no. #29837‐4.

**Table 1:** Location of eight red‐bellied piranha populations in Solimões‐Amazonas River, Brazil

Location	Code	Latitude (S)	Longitude (W)	Sample size
Praia	PRA	−3.209425	−59.896926	30
Camboa	CAM	−3.201798	−59.559440	30
Reis	REI	−3.151750	−59.695396	30
Catalão	CAT	−3.155607	−59.907054	30
São Tomé	STO	−3.801975	−61.516106	30
Ananá	ANA	−3.530318	−61.568055	30
Araçá	ARA	−3.859774	−62.342484	30
Maracá	MAR	−3.826554	−62.559807	24

Code is short name of lakes in Solimões‐Amazonas River.

### DNA extraction and PCR amplification

PCR amplification and data analysis were performed according to Nascimento et al. (2011) and Santos et al. (2014). DNA extraction method was performed as described by Sambrook et al. (1989): 100 mg of tissue was digested overnight at 37°C in 0.7 mL of lysis buffer (6 M urea, 10 mM Tris‐HCl, 125 mM NaCl, 1% SDS, 10 mM EDTA, pH 7.5). After that, we added 10 *μ*L of proteinase K (Fermentas, Waltham, MA, USA), RNase A (Invitrogen, Waltham, MA, USA), and 1,4‐dithiothreitol (Biosynth^®^ Chemistry and Biology, Rietlistr, Staad, Switzerland). After the digestion, we washed the samples with phenol‐chloroform‐isoamyl alcohol (Invitrogen, Waltham, MA, USA) and isopropanol on ice. The pellet was resuspended in 50 *μ*L DNase‐free H_2_O. We verified the quality of the extracted DNA by electrophoresis in a 1.5% agarose gel using GelRed^®^ (Invitrogen, Waltham, MA, USA) and visualized it using an L‐PIX Molecular Image transilluminator (Loccus Biotecnologia, Cotia, SP, Brazil). We quantified the DNA using a NanoDrop^®^ 2000 spectrophotometer (Thermo Scientific, Waltham, MA, USA).

The *P. nattereri* samples were genotyped using the eight microsatellite loci (*PN1*,* PN2*,* PN3*,* PN5*,* PN6*,* PN7*,* PN11*, and *PN13*) developed by Nascimento et al. (2011). We performed the polymerase chain reactions (PCR) using a 96‐well Veriti^™^ Thermal Cycler (Applied Biosystems, Waltham, MA, USA) in a final reaction volume of 10 *μ*L. Each PCR contained 1.5 *μ*L of genomic DNA (50 ng/*μ*L), 1.0 *μ*L of forward primer (0.4 mM), 1.5 *μ*L of reverse primer (0.4 mM), 1.0 *μ*L 6‐FAM fluorescent M13 primer (Schuelke 2000) (0.4 mM), and 5.0 *μ*L PCR Master Mix 2X (Fermentas, Waltham, MA, USA), which contains Taq DNA polymerase (0.05 U), MgCl_2_ (1.5 mM), and dNTPs (0.4 mM). The PCR cycling conditions were as follows: 1 cycle at 94°C for 3 min; 20 cycles at 94°C for 20 sec, 55–58°C for 20 sec (annealing temperature; see Nascimento et al. 2011), and 68°C for 30 sec; 25 cycles at 94°C for 20 sec, 53°C for 20 sec, and 68°C for 30 sec; and finally, the extension cycle was performed at 72°C for 10 min. We confirmed the amplified PCR products by electrophoresis in a 1.5% agarose gel using GelRed^®^ (Invitrogen, Waltham, MA, USA), and we visualized it using an L‐PIX Molecular Image transilluminator (Loccus Biotecnologia, Cotia, SP, Brazil). The amplified products were genotyped on an ABI 3130xl Genetic Analyzer (Applied Biosystems, Waltham, MA, USA) using the GeneScan Liz‐500 (‐250) standard size (Applied Biosystems, Waltham, MA, USA) to determine fragment length. The alleles were scored based on the consistent pattern of their stutter peaks, and on the peak intensity corresponding to each individual at each locus using GeneMapper v4.0 (Applied Biosystems, Waltham, MA, USA).

### Data analysis

We tested microsatellite data from red‐bellied piranha populations for linkage disequilibrium (LD) and Hardy–Weinberg equilibrium (HWE) using Fstat v2.9.3.2 software (Goudet 2001). We used Bonferroni's correction for multiple comparisons (Rice 1989) at a significance level of 5%. Micro‐checker v2.3 software (van Oosterhout et al. 2004) was used to check for detection of null alleles and for scoring errors in large alleles and stutter peaks. In addition, we calculated the frequency of null alleles using Cervus v3.0.3 software (Kalinowski et al. 2007).

We estimated the allelic diversity by the total number of alleles (TNA), mean number alleles (MNA), number of private alleles (NPA) (run by GDA v1.1 software, Lewis and Zaykin 2000), allelic richness (AR) (run by Fstat v2.9.3.2 software), and number of effective alleles (NEA) calculated by a mathematical model: NEA = 1/(1−*H*_*E*_). We estimated the genetic diversity by the observed (*H*_*O*_) and expected heterozygosity (*H*_*E*_) in Hardy–Weinberg equilibrium, fixation index (*F*_*IS*_) (run by Genetix v4.05.2 software, Belkhir et al. 2004), polymorphism information content (PIC) (run by Cervus v3.0.3 software), internal diversity (ID), and gain/loss of genetic diversity (run by Molkin v3.0 software, Gutiérrez et al. 2005).

The levels of genetic differentiation in red‐bellied piranhas populations were analyzed using Wright's *F*‐statistics (*F*_*IT*_, *F*_*IS*_ and *F*_*ST*_, Weir and Cockerham 1984) run by Arlequin v3.5 software (Excoffier and Lischer 2010). The additional analysis of the differentiation indices utilized the following: *F*_*ST*_, which assumes the Infinite Allele Model (IAM, Kimura and Crow 1964); *R*_*ST*_ (Slatkin 1995) which assumes the Stepwise Mutation Model (SMM, Kimura and Otha 1978) (run by Arlequin v3.5 software); and *D*_*ST*_ (Nei 1973), which assumes a total differentiation between populations (run by Smogd v1.2.5 software, Crawford 2010). We calculated the number of migrants (Nm) between populations by applying the *F*_*ST*_ values using the Arlequin v3.5 software. We analyzed the molecular variance (AMOVA, Excoffier and Slatkin 1995) in red‐bellied piranha populations using the Arlequin v3.5 software at the significance level of 5%.

**Figure 3:**
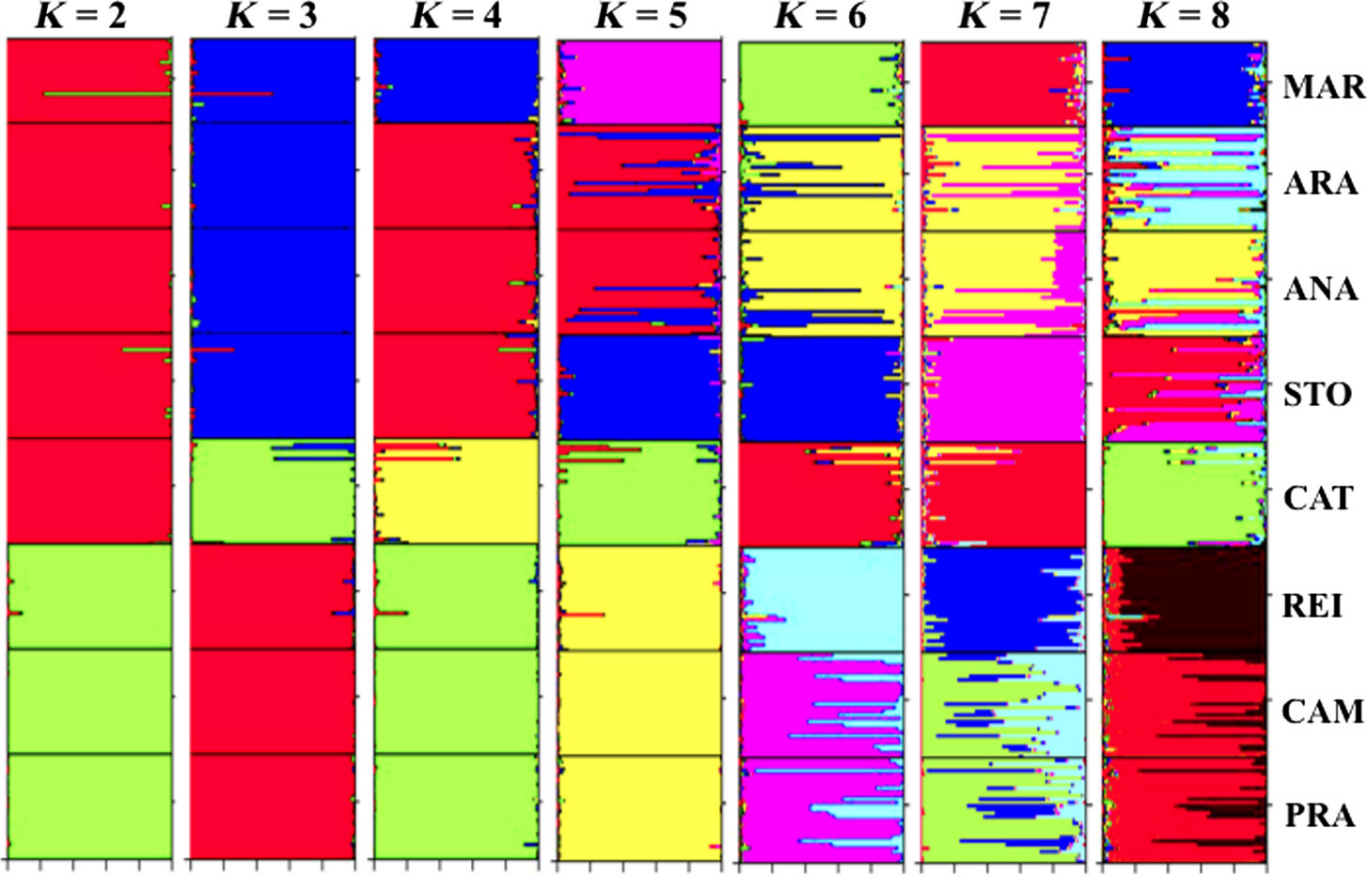
Diagrams showing the identification of genetic structure of red‐bellied piranha populations from the Solimões‐Amazonas River System, including eight lakes. From left to right, increasing values for K show the higher structure of Catalão lake (CAT) subpopulation (see text for explanation). The best results of Delta‐K (Table S1 and Fig. S1) suggest the existence of two biological populations (K = 2) for the Solimões‐Amazonas river: population A (green) and population B (red), respectively, in the first diagram from left to right. The genetic structure found in software Structure were also confirmed by the UPGMA tree (Fig. 5) and FCA (Fig. 6).

Isolation by distance (IBD) over the distribution area was assessed by testing the correlation between genetic and geographical distances considering all population pairs in using the regression of *F*_*ST*_/(1 − *F*_*ST*_) estimates on distance for populations, as suggested by Rousset (1997). This model was tested using Mantel's tests (Mantel 1967). For geographical distances, we considered straight‐line distances between all pairs of sampling sites. IBD was also tested in each cluster of populations identified according to the results of the clustering analysis (see Fig. 3, K = 2) with the same procedure. All these tests and calculations were performed with IBDWS v3.23 (Jensen et al. 2005).

While analyzing the populations’ structure, we applied the mixture model (Admixture) ancestor, which correlates with the gene frequency among the studied populations, using Structure v2.3.1 software (Pritchard et al. 2000; Falush et al. 2003), with a burn‐in of 50,000 followed by 200,000 steps using the Markov chain Monte Carlo's method (MCMC). We applied the value of Δ*K* (suggested by Evanno et al. 2005) to identify the highest level of genetic division hierarchy, which divided the populations and repeated the analysis for each division. Each analysis was performed using K = 1 and K = (number of populations + 2), with five replicates for each K. To complement the analysis of the population structure, we performed a factorial correspondence analysis (FCA, Benzecri 1973) using Genetix v4.05.2 software (Belkhir et al. 2004), which assumes a genetic distance between populations. A UPGMA tree was built based on the genetic distance (*D*_*A*_) using GDA v1.1 software (Lewis and Zaykin 2000).

## Results

A total of 388 alleles were found in the eight subpopulations of red‐bellied piranha distributed among eight microsatellite loci. An overall average of 48.5 allele/population was observed, and the variation of alleles between subpopulations was 39 alleles to fish from lake MAR and 58 alleles to fish from lake CAM. The number of effective alleles was 2.838 (with variation of 2.058–3.559) (Table 2). We found a total of 44 private alleles, with one private allele in fish of lake ANA and 14 private alleles in fish lake CAM. However, we did not find any private allele in the subpopulation of lake ARA (see Table 2), which indicates the distribution or share of all alleles with other subpopulations.

**Table 2:** Genetic diversity indices in red‐bellied piranha populations based on eight microsatellite loci

Populations	*N*	Allelic diversity					Genetic diversity					
		TNA	MNA	NEA	AR	NPA	*H*_*O*_	*H*_*E*_	PIC	*F*_*IS*_	loss/gain	ID
PRA	30	57	7.125	3.559	6.416	4	0.843	0.719	0.699	−0.176	1.616	1.687
CAM	30	58	7.250	3.436	6.340	14	0.725	0.709	0.681	−0.063	1.568	1.262
REI	30	45	5.625	2.967	5.111	4	0.716	0.663	0.638	−0.082	0.359	0.375
CAT	30	50	6.250	2.703	5.678	13	0.668	0.630	0.623	−0.063	2.648	0.263
STO	30	49	6.125	2.857	5.451	4	0.741	0.650	0.639	−0.141	0.209	0.184
ANA	30	45	5.625	2.058	4.941	1	0.477	0.514	0.481	0.072	−1.938	−2.431
ARA	30	45	5.625	2.525	5.148	–	0.571	0.604	0.559	0.055	−1.328	−0.739
MAR	24	39	4.875	2.597	4.674	4	0.504	0.615	0.599	0.183	−0.002	−0.585

N, samples size; TNA, total number of alleles; MNA, mean number of alleles; NEA, number of affective alleles; AR, allelic richness; NPA, number of private alleles; *H*_*O*_, observed heterozygosity; *H*_*E*_, genetic diversity (expected heterozygosity in Hardy–Weinberg equilibrium); PIC, polymorphism information content; *F*_*IS*_, inbreeding coefficient of population; Loss(−)/gain(+), loss or gain genetic and ID, internal diversity.

The results showed the absence of null alleles in all populations, and this information was confirmed when we found the frequency values of possible null alleles in Cervus (value frequency of null alleles < 0.1) (Table 3). In addition, we did not find any deviation from either the Hardy–Weinberg equilibrium (HWE) or the linkage disequilibrium (LD) in microsatellite loci after Bonferroni's correction, showing that these markers have an independent distribution.

**Table 3:** Values of the genetic differentiation indices in red‐bellied piranha populations based on eight microsatellite loci

Loci	Na	*H*_*E*_	*H*_*O*_	PIC	*F*_*IS*_	*F*_*IT*_	*F*_*ST*_	*R*_*ST*_	*D*_*ST*_	*F* (Null)
PN 1	11	0.590	0.535	0.503	0.093*	0.147*	0.054*	0.045*	0.027*	0.049
PN 2	12	0.578	0.573	0.552	0.008	0.207*	0.199*	0.406*	0.104*	0.003
PN 3	17	0.835	0.745	0.815	0.108*	0.319*	0.211*	0.793*	0.155*	0.059
PN 5	18	0.792	0.743	0.768	0.062*	0.331*	0.269*	0.843*	0.191*	0.028
PN 6	19	0.790	0.652	0.762	0.175*	0.297*	0.122*	0.214*	0.085*	0.076
PN 7	21	0.898	0.704	0.887	0.217*	0.374*	0.157*	0.521*	0.133*	0.094
PN 11	18	0.868	0.646	0.852	0.256*	0.601*	0.345*	0.259*	0.280*	0.093
PN 13	13	0.854	0.752	0.854	0.120*	0.291*	0.171*	0.223*	0.134*	0.058

Na, number alleles by loci; *H*_*E*_, genetic diversity (expected heterozygosity in Hardy–Weinberg equilibrium); *H*_*O*_, observed heterozygosity; PIC, polymorphism information content; *F*_*IS*_, fixation index of the subpopulations; *F*_*IT*_, fixation index of the total population; *F*_*ST*_, fixation index result for the comparison of the populations to the total populations; *R*_*ST*_, analogous to *F*_*ST*_ of microsatellites markers; *D*_*ST*_, analogous to *F*_*ST*_ and *F*(null), frequency of null alleles; level of significance, *P < 0.05.

Populations of red‐bellied piranhas showed high genetic variability (*H*_*O*_: 0.504 ‐ 0.843), and (*H*_*E*_: 0.514–0.719) (Table 2). Considering all microsatellite loci, the *H*_*E*_ and *H*_*O*_ values ranged from 0.578 to 0.898 and from 0.535 to 0.752, respectively. The average PIC was 0.615, with variation of 0.503 (*PN1*) to 0.881 (*PN7*) (Table 3). The inbreeding coefficient (*F*_*IS*_) ranged from −0.176 (lake PRA) to 0.183 (lake MAR). Only the subpopulation of lake MAR presented *F*_*IS*_ values above 0.1. We also identified that the subpopulations of the lakes ANA, ARA, and MAR showed lack of heterozygotes, with a trend for loss of genetic variability in these subpopulations.

The indices of genetic differentiation of subpopulations of the analyzed species showed significantly different values (*P < *0.05) for microsatellite loci (Table 3), analysis of molecular variance – AMOVA (Table 5), and pairwise of *F*_*ST*_ (Table 4). We found high *F*_*ST*_ values in pairwise when compared to subpopulation of the lake CAT with other subpopulations analyzed, and smaller *F*_*ST*_ values in pairwise within each cluster or biological populations (Table 4 and Fig. 3). Gene flow was higher within the biological populations, but lower between clusters formed for the red‐bellied piranha populations (See Table 4). However, gene flow was higher in the lakes nearby Manaus because they do have greater connectivity (PRA, CAM, and REI).

**Table 4:** Pairwise population Nm values based on *F*_*ST*_ (above diagonal) and pairwise *F*_*ST*_ values (below diagonal) for red‐bellied piranha populations based on eight microsatellite loci

Lagos	PRA	CAM	REI	CAT	STO	ANA	ARA	MAR
PRA	–	9.49	3.70	0.73	0.94	0.76	0.93	0.95
CAM	**0.022**	–	3.90	0.69	0.91	0.73	0.89	0.87
REI	**0.071**	**0.059**	–	0.66	1.01	0.74	0.93	0.90
CAT	**0.219**	**0.239**	**0.258**	–	0.85	0.84	0.90	0.76
STO	**0.109**	**0.096**	**0.073**	**0.220**	–	1.19	1.78	1.29
ANA	**0.223**	**0.175**	**0.160**	**0.297**	**0.063**	–	2.30	0.86
ARA	**0.130**	**0.098**	**0.082**	**0.223**	**0.058**	**0.049**	–	1.78
MAR	**0.151**	**0.170**	**0.165**	**0.319**	**0.065**	**0.215**	**0.058**	–

Bold data show statistically significant differences (*P* < 0.05; *P* = 0.001786 Bonferroni correction). Dashed lines indicate gene flow between populations within of biological population (above of one individual/generation).

**Figure 4:**
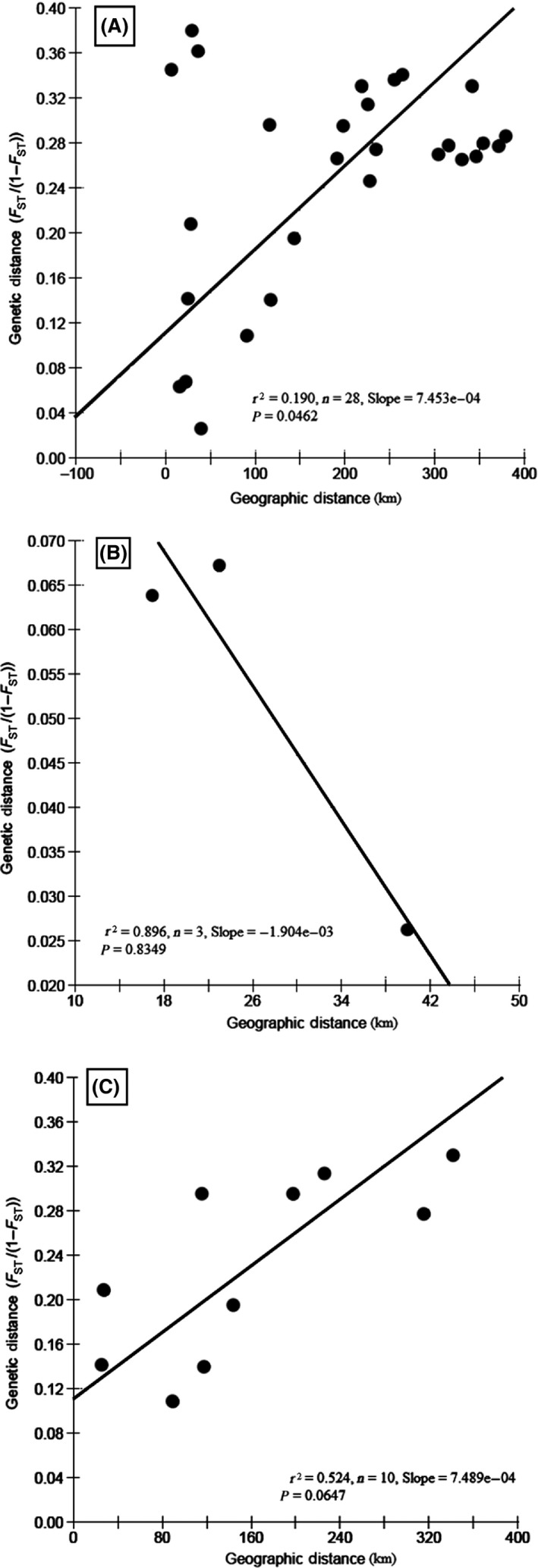
Isolation by distance (IBD) pattern in the distribution area. Relationships between genetic and geographic distance among the eight red‐bellied piranha populations. (A) Positive correlation between genetic [estimated by *F*_*ST*_/(1 − *F*_*ST*_)] and geographical distances (km) for all pairs of sampled populations. (B) Negative correlation between genetic and geographical distance (km) for the pairs of PRA, CAM, and REI subpopulations. (C) Positive correlation between genetic and geographical distance (km) for the pairs of CAT, STO, ANA, ARA, and MAR subpopulations (see Fig. 3 for the definition of these groups).

Isolation by distance data (IBD) shows that the geographical distance between the lakes located at the Solimões‐Amazonas River systems has strong influence on the red‐bellied populations (Fig. 4). However, subpopulation from lake CAT shows divergence from IBD data, suggesting that this subpopulation may be locally adapted (Table S2).

**Figure 5:**
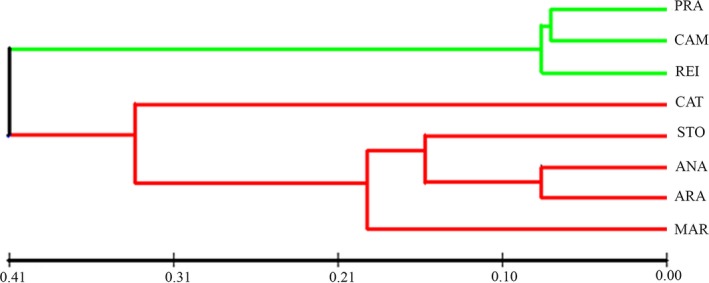
Representation of UPGMA tree for red‐bellied piranha populations in the lakes of Solimões‐Amazonas River. Red‐bellied piranhas’ populations are divided in two biological populations of the Solimões‐Amazonas River (Fig. 3); however, red‐bellied piranha populations of CAT lake (Rio Amazonas) showed greater genetic similarities with the populations of lakes in the Solimões River.

**Figure 6:**
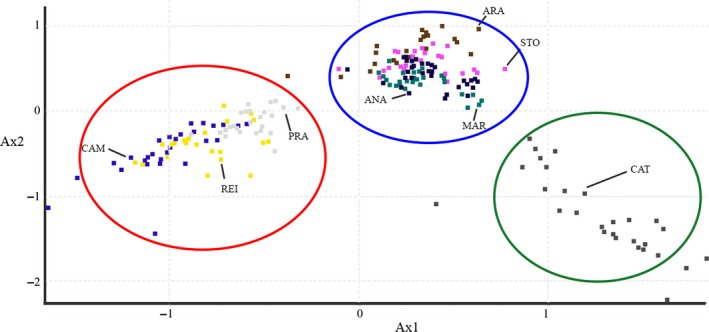
Factorial component analysis (FCA) shows the distribution of spatial variation in red‐bellied piranhas’ populations for lakes in the Solimões‐Amazonas River System. The quarters right (Ax1 > 0) shows the piranhas populations in the STO, ANA, ARA, MAR, and CAT lakes forming a genetically distinct group, and the quarters left (Ax1 < 0) show another genetic group for the PRA, REI, and CAM lakes, respectively. The formation of these two biological populations is also found in the analysis of structure (Fig. 3) and UPGMA tree (Fig. 5; by software GDA). The above (AX2 > 0) and below quarters (AX2 < 0) shows the spatial distribution of the red‐bellied piranha populations from the geographical location (longitudinal), which represent the areas of collections of populations in the natural environment for the Solimões‐Amazon River (Fig. 2 and Table 1).

The structure was used for clustering of individuals in 2 ≤ K ≤ 8 software; however, the lower value of K (K = 2) was the better explanation for the subpopulations of red‐bellied piranha, joining fish from the lakes PRA, CAM, and REI, which formed an independent cluster. In K = 3 to K = 8, the subpopulation of the lake CAT resulted in a separated subpopulation, forming an isolated cluster data set in the cluster diagram of K = 3. In fact, for this population, the great the K, the great was the isolation however, considering all subpopulations. The higher ΔK was K = 2 (see Fig. 3, Table S1 and Fig. S1). Similar data have been found in the UPGMA analysis, as well as in the FCA analysis, where one may see the high divergence from the subpopulation of lake CAT (Figs. 5 and 6). These data can be compared with K analysis, where K = 3 and K = 8 are consistent with the existence of one divergent subpopulation at Catalão lake (CAT). Finally, the Bayesian inference analysis suggests the existence of two biological populations of red‐bellied piranhas in the Solimões‐Amazonas River system (Fig. 3, Table S1 and Fig. S1): one biological subpopulation in the lakes of Rio Solimões basin and another biological subpopulation in the located at Rio Negro. Catalão lake (CAT) is the single place located right in the meeting of these two rivers, where black and white water mixture, completely changing their physicochemical parameters.

## Discussion

Considering the data obtained herein, we can affirm that the genetic variation in red‐bellied piranha populations is strongly related to a subdivision into two biological populations (Table 2). Firstly, we documented a pattern of higher genetic variation in the subpopulations of the lakes PRA, CAM, and REI located at Rio Amazonas, which largely reflects their geographical configuration. There, population differentiation was generally high and remarkably suggested a genetic signature of the individual subpopulations exceeding the signature of population clusters on single lakes. Secondly, lower genetic variation was detected in the subpopulations of the lakes CAT, STO, ANA, ARA, and MAR located at Rio Solimões (red cluster; Figs. 3, S1 and Table S1, K = 2) compared to the subpopulations of the lakes PRA, CAM, and REI (green clusters; Figs. 3, S1 and Table S1, K = 2). We have identified loss of genetic diversity only in the subpopulations of the Solimões lakes ANA, ARA, and MAR, suggesting a long‐term isolation of such populations (Table 2). Our findings shed light on the relationship between genetic variation and geographic distance for this species.

Escobar et al. (2015) reported that *Piaractus brachypomus* (Serrasalmidae) populations inhabiting the Orinoco basin and the Amazon basin had similar allelic diversity; however, the heterozygosity levels were reduced in populations of the Orinoco. Moreover, Santos et al. (2007) found a high genetic variability in *Colossoma macropomum* (Characiforms) populations living in the Rio Amazonas channel, but no genetic differentiation between them, suggesting that this species, which also belongs to Serralmidae family, compose a single panmictic population. Gomes et al. (2013), investigating *Salminus brasiliensis* (Characiforms) populations in Paranapanema River basin, observed high levels of genetic variability using RAPD markers, and high levels of genetic structure in its populations. These results suggest that the species belonging to the Order Characiformes have high genetic variability; however, a decrease in gene flow can induce differentiation between populations. According to Solé‐Cava (2001), the typical wild population presents high values of genetic variability. However, we found the opposite in this study, mainly for some subpopulations from rio Solimões lakes.

In fact, former cytogenetic studies described karyotypic variations between red‐bellied piranhas populations collected in different basins (Nakayama et al. 2008; Santana et al. 2011). The present results show structuring patterns that result in subpopulations collected from the same basins, suggesting the existence of barriers that impair gene flow among them. Therefore, we suggest that the subpopulations of red‐bellied piranhas cannot be considered panmictic, and that further management of these populations must consider their genetic characteristics that suggest different specific genetic stocks.

The present data also show high genetic differentiation among red‐bellied piranha populations in the Solimões‐Amazonas River systems (Tables 4 and 5). Actually, the geographical distance between the lakes causes such high genetic differentiation, which is resulted from a decrease in gene flow among populations. The identification of a significant IBD pattern does not necessarily imply the absence of sharp discontinuities in gene frequencies, and the identification of IBD can help to show equilibrium between migration and genetic drift (contemporary processes), or to link limited dispersal ability and genetic differentiation (Garnier et al. 2004).

**Table 5:** Analysis of molecular variance (AMOVA) for red‐bellied piranha populations based on eight microsatellite loci

Source of variation	Sum of squares	Variance components	Percentage variation	*P*‐value
Within individuals	581.500	2.675	84.116	0.000*
Between individuals	506.625	−0.122	−3.832	1.000
Between population	255.093	0.627	19.717	0.000*
Total	1343.218	3.181	–	–

a Indicates a significant difference at *P* < 0.05.

Considering isolation of populations among the lakes during low water season, there is a reduction in the connection between the most distant lakes; such that the gene flow is limited between the two biological populations, and the geographical distance has a strong influence on the migration of the species between the lakes.

In addition, we can suggest that the flood pulses (Junk et al. 1989; Bittencourt 1994) did not contributed enough to maintain gene flow among these subpopulations due to the large geographical distance between some lakes. In contrast, we found high levels of gene flow in the three lakes located nearby Manaus, and this fact is due to the higher connectivity between the lakes (Table 4), which, coincidently, are located in rio Amazonas (when Rio Negro have already entered Rio Solimões, forming Rio Amazonas), where water quality is completely different from that of Rio Solimões.

Aguirre et al. (2013) observed that populations of *Hoplias microlepis* (Characiformes) have low genetic differentiation within populations of rivers or artificial dams, but present high genetic differentiation between these rivers and the artificial dams. Santos et al. (2007) detected lower genetic differentiation among *C. macropomum* populations in the rio Amazonas, while Farias et al. (2010) detected high genetic differentiation among *C. macropomum* populations living both in the Amazon basin and in the subbasin of Bolivia.

The present data corroborate the results obtained by Santos et al. (2007), Farias et al. (2010), and Aguirre et al. (2013) that clearly showed that the populations of Characiformes tend to have higher genetic differentiation whenever there are barriers that disrupt gene flow. Merila and Crnokrak (2001) and McKay and Latta (2002) suggested that the quantification of genetic differentiation degrees between subpopulations may be the result of traits under strong divergent selection, and that high *F*_*ST*_ values are expected. Moreover, these authors also mention that this is one of the ways to identify traces of local adaptation.

The adaptive evolutionary plasticity is another alternative for local adaptation and can be manifested as a reduction of the phenotypic difference between demes living in different habitats (Sultan and Spencer 2002). However, high rate of gene flow between subpopulations has the ability to reduce the presence of locally adapted residents (Kawecki and Ebert 2004). The premise to result in local adaptation involves little or absence of gene flow between these subpopulations (Fraser et al. 2001).

Our results have shown strong evidence of local adaptation to the subpopulation of lake CAT and this is reinforced when we notice the high frequency of private alleles (Table 1), high divergence in the analysis of UPGMA and FCA (Figs. 5 and 6), low gene flow and high *F*_*ST*_ values in pairs between subpopulations (Table 4).

As above‐mentioned, the region of lake Catalão corresponds to an environment marked by the beginning of mixed waters of the Rio Solimões (white water) with the Rio Negro (black water) and may have induced local adaptations in the fish living in that kind of watercourses.

Instead, the subpopulations gathered in geographical clusters around Manaus live in a mixed water environment (Rio Amazonas) while the other clusters live in a solely water environment (Rio Solimões), which totally differs from the patterns found in the lake CAT subpopulation from white water clusters. Another interesting fact in this study is that the CAT specimens do not show any phenotypic trait that could distinguish this subpopulation from the other, showing strong correlation with the results described by Sultan and Spencer (2002) for local adaptation.

Structure data, associated with the K‐Delta value (Fig. S1 and Table S1), suggest the existence of two red‐bellied piranha biological populations in the Solimões‐Amazonas River (see Figs. 3, 5, and 6). Vicentin et al. (2013) reported that differences between populations might require different strategies for the management of fish resources. According to Escobar et al. (2015), the occurrence of distinct populations (Evolutionary Significant Unit, ESU) suggests a requirement for independent management of fishing among the stocks, and that any translocation of stocks by fishing between different populations should be avoided, so no loss of local adaptation or extinction associated with exogamous depression would happen. In this context, we suggest that any resolution that aims to regulate fishing of red‐bellied piranhas in the Solimões‐Amazonas River systems should take into consideration the existence of two biological populations in the study area.

In conclusion, our results clearly show that the populations located nearby Manaus and in the Varzea Lake's communities might be susceptible to human actions such as environmental pollution and overfishing. Once we observed signs of population expansion in red‐bellied piranha populations nearby Manaus city, verified through the NPA's higher values in red‐bellied piranha populations from the lakes PRA, CAM, REI, and CAT (see values of NPA, Table 2), we believe that these subpopulations may be under conservation programs as a permanent source of genetic variability. Thus, the creation of a reserve in the surrounding area of these lakes would be an important initiative to restrict anthropic damages to these populations. This action would protect the piranha populations in the region and prevent environmental changes caused by man, avoiding affect genetic health of these piranha populations, which are valuable aquatic resource for food chain in nature, and for human protein access.

## Conflict of Interest

The authors declare no conflict of interest.

## Supporting information


**Figure S1.** Inference of best K using Delta‐K values (Evanno et al. 2005) shows the rate of change between successive values of K such that a peak value is interpreted as the “true” K.

**Table S1.** Evanno table output for Structure run with all populations.
**Table S2.** Rousset (1997) distance (*F*_*ST*_ / 1 − *F*_*ST*_) (above diagonal) and similarity (*M* = (1/*F*_*ST*_ − 1)/4) by Stalkin's (1993) (below diagonal) for red‐bellied piranha populations based on eight microsatellite loci.
