# Supplementary figures and images for: Genetic differentiation in red‐bellied piranha populations (Pygocentrus nattereri, Kner, 1858) from the Solimões‐Amazonas River
Source: Ecol Evol. 2016 May 24;6(12):4203–13. doi: 10.1002/ece3.2195 (PMC4972243; doi:10.1002/ece3.2195)

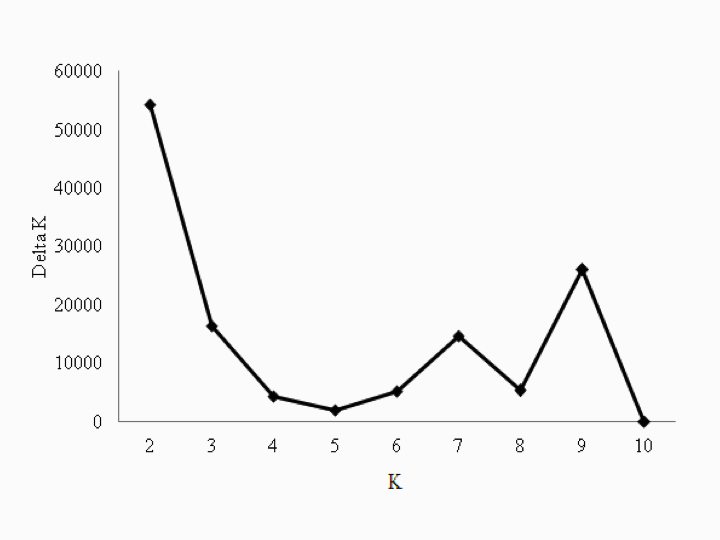

Supplement: Supplementary file 1 — Figure S1. Inference of best K using Delta‐K values (Evanno et al. 2005) shows the rate of change between successive values of K such that a peak value is interpreted as the “true” K. [file ECE3-6-4203-s001.tiff]
