# Supplementary material for: Genetic differentiation in red‐bellied piranha populations (Pygocentrus nattereri, Kner, 1858) from the Solimões‐Amazonas River
Source: Ecol Evol. 2016 May 24;6(12):4203–13. doi: 10.1002/ece3.2195 (PMC4972243; doi:10.1002/ece3.2195)
Supplement: Supplementary file 2 — Table S1. Evanno table output for Structure run with all populations. Table S2. Rousset (1997) distance (F ST / 1 − F ST) (above diagonal) and similarity (M = (1/F ST − 1)/4) by Stalkin's (1993) (below diagonal) for red‐bellied piranha populations based on eight microsatellite loci. [file ECE3-6-4203-s002.docx]

Table S1.

| K | Reps | L(K) | Stdev | L’(K) | L’’(K) | [L’’(K)] | Delta K |
| --- | --- | --- | --- | --- | --- | --- | --- |
| 1 | 5 | -6759.0 | 7.84219 | - | - | - | - |
| **2** | **5** | **-5769.4** | **10.90871** | **989.2** | **-591.8** | **591.8** | **54.25023** |
| 3 | 5 | -5372.4 | 13.00769 | 397.4 | -212.7 | 212.7 | 16.35186 |
| 4 | 5 | -5187.7 | 14.54648 | 184.7 | -062.1 | 062.1 | 4.26907 |
| 5 | 5 | -5065,1 | 15.99687 | 122.6 | -030.3 | 030.3 | 1.89412 |
| 6 | 5 | -4972,8 | 18.20440 | 092.3 | -093.5 | 093.5 | 5.13744 |
| 7 | 5 | -6196,8 | 53.31885 | -1.244 | 777.1 | 777.1 | 14.57503 |
| 8 | 5 | -5420.9 | 38.33667 | 775.9 | -201.2 | 201.2 | 5.24824 |
| 9 | 5 | -4846.2 | 22.35397 | 574.7 | -576.6 | 576.6 | 25.92815 |
| 10 | 5 | -6743,2 | 65.52633 | -1.897 | -001.9 | 001.9 | 0.02895 |

Table S2.

| Lagos | PRA | CAM | REI | CAT | STO | ANA | ARA | MAR |
| --- | --- | --- | --- | --- | --- | --- | --- | --- |
| PRA | ----- | **0.022(40km)** | **0.076(23km)** | 0.280(7km) | 0.122(191km) | 0.287(219km) | 0.149(305km) | 0.178(331km) |
| CAM | 11.114 | ----- | **0.063(17km)** | 0.314(37km) | 0.106(235km) | 0.212(263km) | 0.109(353km) | 0.205(379km) |
| REI | 3.271 | 3.987 | ----- | 0.348(30km) | 0.079(228km) | 0.190(256km) | 0.089(346km) | 0.198(372km) |
| CAT | 0.891 | 0.796 | 0.719 | ----- | 0.282(198km) | 0.422(226km) | 0.287(316km) | 0.468(342km) |
| STO | 2.044 | 2.354 | 3.174 | 0.886 | ----- | **0.067(28km)** | **0.062(118km)** | **0.052(144km)** |
| ANA | 0.871 | 1.178 | 1.312 | 0.592 | 3.718 | ----- | **0.052(90km)** | 0.274(116km) |
| ARA | 1.673 | 2.301 | 2.799 | 0.871 | 4.060 | 4.852 | ----- | **0.062(26km)** |
| MAR | 1.405 | 1.220 | 1.265 | 0.534 | 3.596 | 0.913 | 4.060 | ----- |

Bold data indicate low values of IBD (isolation by distance) between subpopulations within of biological population. Values within the parentheses represent the geographical distance (km) approximate between the lakes of this study.

**Reference**

Evanno G, Regnaut S, Goudet J (2005) Detecting the number of clusters of individuals using the software Structure: a simulation study. *Molecular Ecology*, **14**, 2611-2620.

Rousset F (1997) Genetic differentiation and estimation of gene flow from F-statistics under isolation by distance. *Genetics*, **145**, 1219-1228.

Slatkin M (1993) Isolation by distance in equilibrium and non-equilibrium populations. *Evolution*, **47**, 264-279.
